# Cluster-randomized controlled trial of the effects of free glasses on purchase of children's glasses in China: The PRICE (Potentiating Rural Investment in Children's Eyecare) study

**DOI:** 10.1371/journal.pone.0187808

**Published:** 2017-11-21

**Authors:** Xiuqin Wang, Nathan Congdon, Yue Ma, Min Hu, Yuan Zhou, Weiqi Liao, Ling Jin, Baixiang Xiao, Xiaoyi Wu, Ming Ni, Hongmei Yi, Yiwen Huang, Beatrice Varga, Hong Zhang, Yongkang Cun, Xianshun Li, Luhua Yang, Chaoguang Liang, Wan Huang, Scott Rozelle, Xiaochen Ma

**Affiliations:** 1 State Key Laboratory of Ophthalmology and Division of Preventive Ophthalmology, Zhongshan Ophthalmic Center, Sun Yat-sen University, Guangzhou, Guangdong, China; 2 Affiliated Hospital of Guangdong Medical University, Zhanjiang, Guangdong, China; 3 Orbis International, New York, New York, United States of America; 4 Translational Research for Equitable Eyecare, Center for Public Health, Queen’s University Belfast, Belfast, N. Ireland; 5 The Center for Experimental Economics in Education, Shaanxi Normal University, Xi'an, Shaanxi, China; 6 The Second People's Hospital of Yunnan Province, Kunming, Yunnan, China; 7 The Fred Hollows Foundation China, Kunming, Yunnan, China; 8 Center for Chinese Agricultural Policy, Institute of Geographical Sciences and Natural Resources Research, Chinese Academy of Sciences, Beijing, China; 9 The Fred Hollows Foundation Australia, Sydney, Australia; 10 The First People’s Hospital of Honghe Prefecture, Yunnan, China; 11 Dehong Prefecture People’s Hospital, Yunnan, China; 12 Chuxiong Prefecture People's Hospital, Yunnan, China; 13 Jianchuan County People’s Hospital, Yunnan, China; 14 The First People's Hospital of Lancang County, Yunnan, China; 15 Freeman Spogli Institute of International Studies, Stanford University, Palo Alto, California, United States of America; 16 China Center for Health Development Studies, Peking University, Beijing, China; Universidade do Minho, PORTUGAL

## Abstract

**Background:**

Offering free glasses can be important to increase children’s wear. We sought to assess whether “Upgrade glasses” could avoid reduced glasses sales when offering free glasses to children in China.

**Methods:**

In this cluster-randomized, controlled trial, children with uncorrected visual acuity (VA)< = 6/12 in either eye correctable to >6/12 in both eyes at 138 randomly-selected primary schools in 9 counties in Guangdong and Yunnan provinces, China, were randomized by school to one of four groups: glasses prescription only (Control); Free Glasses; Free Glasses + offer of $15 Upgrade Glasses; Free Glasses + offer of $30 Upgrade Glasses. Spectacle purchase (main outcome) was assessed 6 months after randomization.

**Results:**

Among 10,234 children screened, 882 (8.62%, mean age 10.6 years, 45.5% boys) were eligible and randomized: 257 (29.1%) at 37 schools to Control; 253 (28.7%) at 32 schools to Free Glasses; 187 (21.2%) at 31 schools to Free Glasses + $15 Upgrade; and 185 (21.0%) at 27 schools to Free Glasses +$30 Upgrade. Baseline ownership among these children needing glasses was 11.8% (104/882), and 867 (98.3%) children completed follow-up. Glasses purchase was significantly less likely when free glasses were given: Control: 59/250 = 23.6%; Free glasses: 32/252 = 12.7%, P = 0.010. Offering Upgrade Glasses eliminated this difference: Free + $15 Upgrade: 39/183 = 21.3%, multiple regression relative risk (RR) 0.90 (0.56–1.43), P = 0.65; Free + $30 Upgrade: 38/182 = 20.9%, RR 0.91 (0.59, 1.42), P = 0.69.

**Conclusions:**

Upgrade glasses can prevent reductions in glasses purchase when free spectacles are provided, providing important program income.

**Trial registration:**

ClinicalTrials.gov Identifier: NCT02231606. Registered on 31 August 2014.

## Introduction

Under-corrected refractive error (URE) accounts for 90% of visual disability among rural Chinese children [1–6], and among the 13 million children in the world visually impaired from URE, some half live in China [7]. Despite the fact that glasses wear is a safe and effective means of correcting refractive error [8], and is associated with significant, trial-proven increases in educational outcomes [9], only 15–20% of Chinese children in rural [9, 10] and urban migrant [11, 12] population studies who need glasses have them.

Various explanations exist for this situation [13]. Many stakeholders, including children, families and teachers, believe incorrectly that wearing glasses will harm children's eyes [14, 15], despite randomized trial evidence to the contrary [8]. The quality of available glasses in rural China is poor: half of children wearing glasses have a power inaccurate by > 1 diopter [16], while two-thirds of rural refractionists practicing in private optical shops have a high school education or less [17].

Cost of glasses also remains a critical barrier: providing free spectacles more than doubled the rates of use 6 months later, compared to providing prescriptions alone [9]. By contrast, educational interventions alone, without free glasses, have been un-successful in increasing rates of wear [18]. However, providing free glasses may not be sustainable. School vision screening programs needed to identify children requiring glasses depends on profits from glasses sales for entities such as rural county hospitals in order to sustain them. Large-scale government programs providing free glasses would be likely to drive such practitioners out of business. A model combining the advantages of free glasses (lack of financial barriers) and glasses sales (sustainability) is needed.

We carried out a cluster-randomized, controlled trial in rural Guangdong and Yunnan Provinces, China, to evaluate the effect of providing “upgrade spectacles” (having stylish designs and scratch-free coatings) on the purchase (main outcome) and wear (secondary outcome) of spectacles among primary school children. We hypothesized that providing free glasses would reduce spectacle purchase, but that the offer of upgrade spectacles for sale at the point of distribution at county hospitals would prevent this reduction, that is, rates of spectacle purchase would not differ between the Control and two Upgrade groups.

## Methods

### Ethics, consent and permissions

This study was approved by the institutional review boards at the Zhongshan Ophthalmic Center (Guangzhou, China) and Yunnan Red Cross Hospital (Kunming, China). Permission was received from local boards of education in each region and the principals of all schools. Written informed consent was obtained from at least one parent of all participating children. The presented data are anonymized and risk of identification is low. The principles of the Declaration of Helsinki were followed throughout.

### Trial design

This was a cluster-randomized, investigator-masked, controlled trial.

### Setting, sampling and eligibility criteria

The study was carried out in Guangdong and Yunnan Provinces, China. Guangdong ranked in the top third (9^th^) among China’s 31 administrative divisions in per capita Gross Domestic Product in 2014 (US$ 10,330), while Yunnan was 29^th^ (US$4438) [19]. We selected 9 counties or county-level cities, five from Yunnan and four from Guangdong, on the basis of having a County-level hospital capable of providing refractive services and willing to participate in the study. All of these hospitals had comparable levels of capacity to refract and dispense glasses due to their current or previous involvement in programs with international non-government development organizations. Spectacles were available for purchase from other, non-study sources in all 9 counties, but no other free spectacle programs were on-going in these counties during the study.

We obtained a list of all 601 primary schools (362 in Guangdong and 239 in Yunnan) in the sample counties from local bureaus of education. Bureau officials also provided information on the number of classes in each school and the number of students per class. For logistical reasons we excluded those schools with average class sizes <20 or >60 students (19% of the sample frame). These criteria were adopted because screening at larger schools could not be reliably completed in a day, which would have interfered with the screening schedule, and smaller schools would be expected to have <7 children requiring glasses, the minimum number required in our power calculations. From the list of 601 schools, we randomly selected 107 (57 schools in Guangdong and 50 in Yunnan), with the number of schools selected in each county being determined by population size.

At the time of our initial screening visit to all schools, we discovered the prevalence of refractive error was lower than expected, requiring an increase in the number of schools in order to achieve adequate power for the study. An additional 31 schools were randomly selected as above, for a total of 138 schools (88 in Guangdong and 50 in Yunnan). Within each sampled school, we randomly selected one class in each of the fourth and fifth grades (likely age range 9–12 years), if there was more than one class per grade level.

### Visual acuity assessment

Children underwent baseline visual acuity screening at school by two trained volunteers. Visual acuity was tested separately for each eye without refraction at four m using early treatment diabetic retinopathy study charts [20] (Precision Vision, La Salle, IL) in a well-lighted, indoor area. If children correctly identified the orientation of at least four of five optotypes on the 6/60 line, they were examined on the 6/30 line, then the 6/15 line, and then line by line to 6/3. We defined visual acuity for an eye as the lowest line on which four of five optotypes were read correctly. If the top line could not be read at four m, the participant was tested at one m and the measured visual acuity was divided by four.

### Refraction

Children with uncorrected visual acuity ≤6/12 in either eye underwent cycloplegia with up to three drops of cyclopentolate 1% in each eye after anesthesia with topical proparacaine hydrochloride 0.5%. Children then underwent automated refraction (Topcon KR 8900, Tokyo, Japan) with subjective refinement by an experienced optometrist. Participating optometrists were from Zhongshan Ophthalmic Center in Guangdong and the Second People's Hospital in Yunnan, both tertiary referral facilities, and all had extensive experience in the refraction of children. Children of parents refusing permission for cycloplegia (274/882 = 31.1%) underwent subjective refinement of the non-cycopleged value from the auto-refractor by an experienced optometrist in each eye using a target at four meters distance.

Children in selected classes were eligible for the study if they met the following criteria:

- Uncorrected (without glasses) visual acuity of ≤6/12 in either eye;
- Refractive error in a range previously demonstrated to be associated with significantly greater improvement with visual acuity when corrected: myopia ≤−0.75 diopters (D), hyperopia ≥2.00 D, or astigmatism (non-spherical refractive error) ≥1.00 D [21];
- Visual acuity could be improved to >6/12 in both eyes with glasses.

### Questionnaires

At baseline (September 2014, beginning of the school year), enumerators administered questionnaires to children, including questions on race (Han versus various minority groups), age, sex, glasses wear, awareness of refractive status, belief that wearing glasses harms children’s vision, parental living condition and education, and ownership of a list of 16 selected items as an index of family wealth. At endline (June 2015, end of the school year), questionnaires were administered on glasses ownership, glasses wear, parental attitude toward wearing glasses and subjective evaluation of project glasses. All questionnaires used in the current study have been validated by our research team in previous publications [8, 9].

### Randomization and interventions

**Fig 1:**
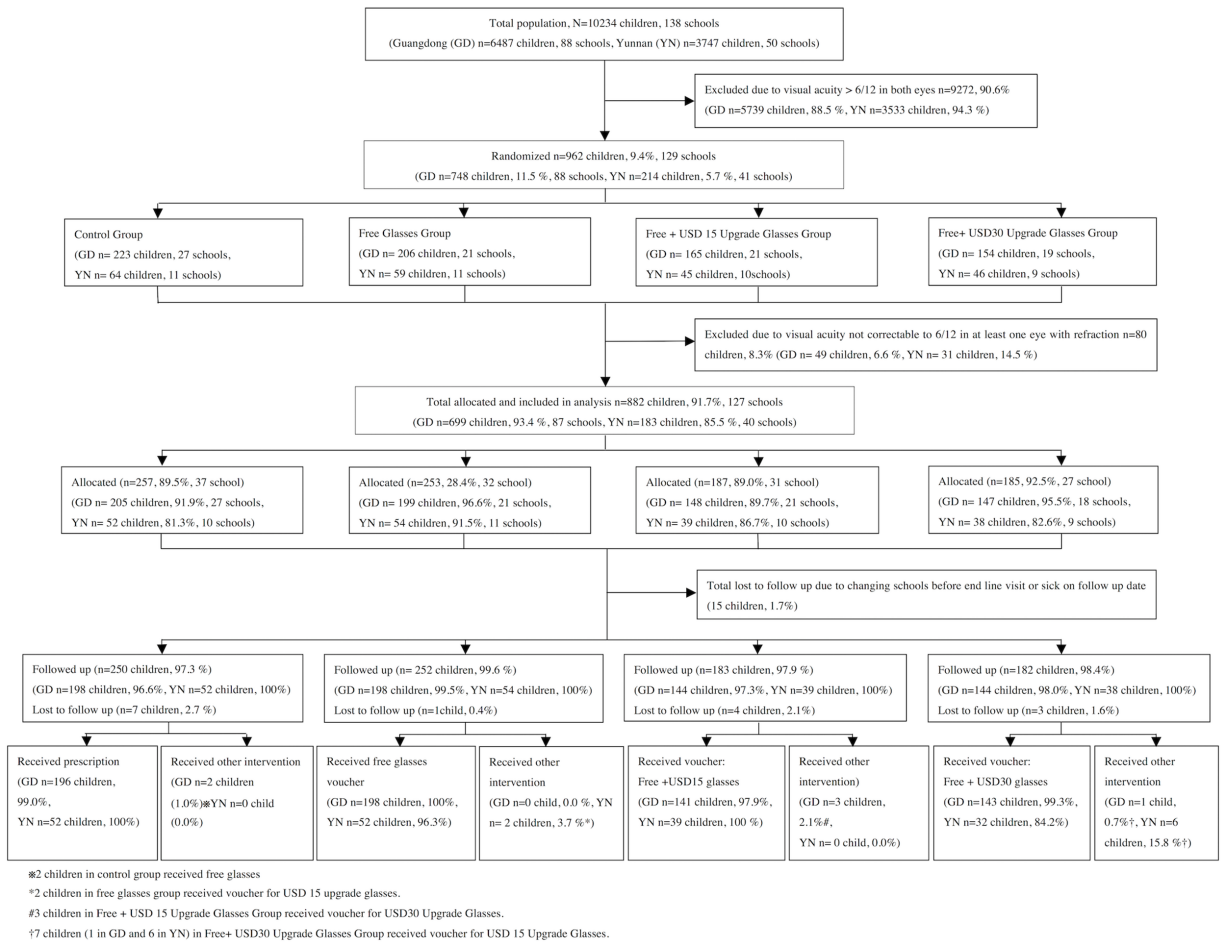
Enrolment and progression of children through the study.

This was a cluster-randomized, controlled trial, with schools as the clusters (Fig 1).

In October 2014, after the baseline survey and vision screening but before refraction, eligible children were randomized by school to receive one of the following four interventions:

- A glasses prescription and letter to the parents informing them of the refractive status of their child. (Control group: 42 schools assigned, 4 schools eliminated in Yunnan due to lack of eligible students, leaving 38 schools.) These children received free spectacles at the end of the trial, although this was not previously announced. Families could elect to purchase glasses at study hospitals or elsewhere if they wished during the study.
- Vouchers bearing the child’s name, school, and refractive power in each eye, exchangeable for free glasses at the local county hospital. (Free group; 32 schools)
- Vouchers exchangeable for free glasses at the local county hospital, where “upgrade glasses” (with scratch-proof lenses and popular designs based on previous research [22] on the style preferences of Chinese children) were also offered for sale to study participants at a price of US$15. (Free + $15 Upgrade group; 32 schools assigned, 1 school eliminated in Yunnan due to lack of eligible students, leaving 31 schools).
- Vouchers for free glasses at the county hospital, where upgrade glasses identical to those above were also offered, at a price of US$30. (Free + $30 Upgrade group; 32 schools assigned, 4 schools eliminated in Yunnan due to lack of eligible students, leaving 28 schools).

The figures of $15 and $30 (25^th^ and 75^th^ percentiles respectively of the amount paid for glasses in a previous study of glasses wear in rural China [9]) were selected as typical for the area. Vouchers were color-coded by study group and valid only at study hospitals, so that families were offered only the spectacle selection appropriate for their allocated study group. Randomization was carried out to allocate all but ten schools. These remaining ten schools were all allocated to the control group, in order to maximize power and minimize the accumulated probability of type II error [23, 24]. Our study hypothesis was that providing free glasses would reduce spectacle purchase, but that the offer of upgrade spectacles would prevent this reduction, that is, rates of spectacle purchase would not differ between the Control and two Upgrade groups.

County hospitals were located at a median distance of 27 km (Guangdong: Range 3–63 km; Yunnan: Range 4–113 km) from the children’s township of residence. Schools were stratified by three variables, information on which was collected during baseline vision screening: county; the total number of students in grades four and five; and the number of students failing vision screening in grades four and five. Stratification and randomization were carried out by an investigator (XM) at a central location (Stanford University, Stanford, CA) using R software (R Foundation for Statistical Computing, Vienna, Austria). Participants (students, parents, and teachers) and enumerators were not informed of either the overall design of the study or the explicit treatment arm assignment. Participants were told only that this was a study of vision care among rural, school aged children.

### Assessment of outcomes

Purchase of glasses (main outcome): Records at the participating county hospitals were used to determine family’s acceptance of free glasses and selection of upgrade glasses (where provided) given by the study within each group. Children’s self-report on questionnaires at the endline examination provided data on purchase of spectacles outside of the study.

Wear of glasses (Secondary outcome): At closeout, spectacle wear was assessed through unannounced direct examinations, with only those children actually wearing their glasses qualifying. Study staff were unaware of the spectacle designs offered in the different groups, and were masked to group assignment.

### Sample size and power

Power calculations were performed using Optimal Design software (http://sitemaker.umich.edu/group-based/optimal_design_software) for cluster- randomization and binary outcome (purchase vs non-purchase, wear vs non-wear). We determined that a sample size of 108 schools (27 per group) with a minimum of seven eligible students per school conferred 80% power, with an α of 0.05, intraclass correlation of 0.15 (based on our previous similar studies), and explained variation by covariates (R2) of 0.50, to detect a difference of 7.5% in eyeglasses purchase and wear rates between intervention arms and the control group. As noted above, the figure of 7 children per school was based on the refractive error prevalence encountered in our previous studies, and the true number per school was closer to 6. We calculated an additional 31 schools were needed to confer the above power under the same assumptions given this smaller number of myopic students per school.

### Statistical methods

Results were presented as mean (SD, standard deviation) for continuous data and frequency (percentage) for categorical data. Baseline wear of glasses was defined as having glasses at school, having been told previously to bring them. We calculated family wealth by summing the value, as reported in the China Rural Household Survey Yearbook (Department of Rural Surveys, National Bureau of Statistics of China, 2013), of household items owned by the family from a previously-defined list of 13 common objects.

Refractive power was defined throughout as the spherical equivalent, that is, the spherical power plus half the cylindrical power. In view of the relatively high rate of parental refusal for cycloplegia, various baseline characteristics were compared between children with and without cycloplegic refraction, using linear regression for continuous variables, logistic regression for binary variables, multinomial logistic regression for nominal categorical variables and ordinal logistic regression for ordinal variables, all with adjustment for cluster effects within schools.

Generalized linear models with Poisson regression were used to estimate the relative risk by intervention arms for purchase and wear of glasses. All variables significant at the p< = 0.2 level in simple regression models were included in the multiple regression model. Regression analyses were performed separately for all children, and only including children undergoing cycloplegic refraction. Statistical analyses were performed using a commercially available software package (Stata 13.1, StataCorp, College Station TX, USA).

To satisfy the requirements of intention to treat analysis that all randomized participants should be included in analyses, we used multiple imputation in Stata to carry out the imputation of missing data [25], selecting the independent variables based on predictive value and availability of data: family wealth (n = 4), having friends wearing glasses (n = 13), purchase of glasses (n = 15) and wear of glasses (n = 15). We used logistic regression for binary variables, ordinal logistic regression for ordinal variables, and multinomial logistic regression for nominal variables. The multiple imputation approach created 20 copies of the data in which missing values were imputed by chained equations [25]. Final results were obtained by averaging these 20 datasets using Rubin’s rules, which ensured that the standard errors for all regression coefficients took into account uncertainty in the imputations, as well as uncertainty in the estimation [25].

## Results

Fig 1 details the enrollment and progression of children through the study. Among 882 children allocated to the four study groups and analyzed here, 608 (68.9%) received parental consent for cycloplegic refraction. Acceptance of cyclopegia was a higher in Yunnan (149/182 = 81.4%) than Guangdong (459/700 = 65.6%, P = 0.01), but children undergoing cycloplegia did not differ significantly by other baseline characteristics, including group assignment, from children who did not (data not shown). Children of families refusing cycloplegia underwent subjective refinement by an experienced refractionist of the power provided by automated refraction, and are included in all analyses.

Table 1 gives the baseline demographic and clinical information, as well as other potential predictors of spectacle purchase and wear, for each of the four study groups. The mean (SD) age among all eligible children was 10.6 (1.0) years, 45.5% (401/882) were boys, and these characteristics varied little across groups. Only 11.8% (104/882) of these children, all of whom would benefit visually from spectacle wear, actually owned glasses at baseline. Refractive error was < = -2.0 D (that is, more myopic than -2.0D) in 36.6% (323/882), and the uncorrected visual acuity was < 6/18 in the better-seeing eye among 35.3% (311/882). Roughly equal numbers of parents had out-migrated to seek work (17.5%, 154/882) and wore glasses (19.5%, 172/882). (Table 1)

**Table 1:** Baseline characteristics of 882 children with correctable refractive error by randomization groups. Values are numbers (percentages) unless stated otherwise.

Characteristics	Total (n = 882)	Control group (n = 257)	Free group (n = 253)	Free + $15 Upgrade group (n = 187)	Free + $30 Upgrade group (n = 185)
Number of school included	129	38	32	31	28
**Individual level variables**					
Age (years) Mean (SD)	10.6 (1.0)	10.5 (0.9)	10.6 (0.9)	10.7 (1.1)	10.6 (1.0)
Male sex	401 (45.5)	117 (45.5)	108 (42.7)	88 (47.1)	88 (47.6)
Owning glasses at baseline*	104 (11.8)	35 (13.6)	35 (13.8)	18 (9.6)	16 (8.7)
Yunnan residence	183 (20.8)	52 (20.2)	54 (21.3)	39 (20.9)	38 (20.5)
Refractive error (Diopters)					
< = −2.00	323 (36.6)	109 (42.4)	92 (36.4)	62 (33.2)	60 (32.4)
> −2 to −0.5 (-2, 0.5]	479 (54.3)	127 (49.4)	139 (54.9)	107 (57.2)	106 (57.3)
> −0.5 to 0.5 (-0.5, 0.5]	60 (6.8)	13 (5.1)	18 (7.1)	13 (7.0)	16 (8.7)
>0.5	20 (2.3)	8 (3.1)	4 (1.6)	5 (2.7)	3 (1.6)
Uncorrected visual acuity <6/18 in eye with better vision	311 (35.3)	89 (34.6)	92 (36.4)	67 (35.8)	63 (34.1)
Only child in family	126 (14.3)	32 (12.5)	41 (16.2)	29 (15.5)	24 (13.0)
One or both parents with ≥12 years of education	272 (30.8)	74 (28.8)	82 (32.4)	63 (33.2)	54 (29.2)
Both parents away from the home the majority of time	154 (17.5)	43 (16.7)	42 (16.6)	36 (19.3)	33 (17.8)
At least one parent wears glasses	172 (19.5)	47 (18.3)	52 (20.6)	43 (23.0)	30 (16.2)
Study time each day after school					
<0.5 hr	341 (38.7)	96 (37.4)	94 (37.1)	84 (44.9)	67 (36.2)
0.5–1 hr	293 (33.2)	83 (32.3)	87 (34.4)	51 (27.3)	72 (38.9)
1–2 hrs	155 (17.6)	48 (18.7)	44 (17.4)	33 (17.7)	30 (16.2)
>2 hrs	93 (10.5)	30 (11.7)	28 (11.1)	19 (10.2)	16 (8.7)
Family wealth ^†^					
Bottom tercile	283 (32.2)	91 (35.6)	69 (27.6)	55 (29.4)	68 (36.8)
Middle tercile	301 (34.3)	82 (32.0)	99 (39.6)	61 (32.6)	59 (31.9)
Top tercile	294 (33.5)	83 (32.4)	82 (32.8)	71 (38.0)	58 (31.4)
**Cluster level variables**					
Grade level (mean number of children per cluster (SD)):					
Fourth grade	3.1 (2.2)	2.9 (2.4)	3.35 (2.1)	2.7 (1.9)	3.6 (2.5)
Fifth grade	4.5 (3.6)	4.6 (3.9)	4.97 (3.3)	4.0 (3.4)	4.6 (4.0)

*Defined as having glasses at school at baseline, having previously been told to bring them to school.

^†^ 4 missing values, 1 in Control group and 3 in Free group

Six month follow up was completed by 867 (98.3%) of children, and no group had lower than 97% follow-up. (Fig 1) The correct intervention as allocated was received by 99.2% (248/250), 99.2% (250/252), 98.4% (180/183) and 96.2% (175/182) of children in the Control, Free, Free + US$15 Upgrade and Free + US$30 upgrade groups.

The proportion of families purchasing glasses (main study outcome) was significantly lower when free glasses were given: Control Group: 59/250 = 23.6%; Free Glasses Group: 32/252 = 12.7%, P = 0.010. Offering Upgrade Glasses eliminated this difference: Free + $15 Upgrade: 39/183 = 21.3%, multiple regression relative risk (RR) 0.90 (0.56–1.43), P = 0.65; Free + $30 Upgrade: 38/182 = 20.9%, RR 0.91 (0.59, 1.42), P = 0.69. (Table 2) Observed spectacle wear at follow-up (Secondary outcome) in the Free Glasses (80/252 = 31.8%, P = 0.07), Free + US$15 (43/183 = 23.5%, P = 0.57) and Free + US$30 (32/182 = 17.6%, P = 0.68) Upgrade groups did not differ significantly from Control (51/259 = 19.7%). (Table 2)

**Table 2:** Number of children needing glasses, owning glasses at baseline, purchasing glasses prior to endline (main study outcome) and observed wearing glasses at endline (secondary outcome), by randomization group.

Characteristics	Control group	Free group	Free + USD15 upgrade glasses group	Free glasses + USD30 upgrade glasses group
Needing glasses* **N**	257	253	187	185
**Owned glasses at baseline N (%)**	35 (13.6)	35 (13.8)	18 (9.6)	16 (8.7)
**Purchased glasses by endline N (%)**^†^	59/250 (23.6)	32/252 (12.7) ^§^	39/183 (21.3)	38/182 (20.9)
**Observed wearing glasses at endline N (%)**^†^^**,**^ ^‡^	50/250 (20.0)	80/252 (31.8)	43/183 (23.5)	32/182 (17.6)

* Defined as having uncorrected visual acuity ≤6/12 in either eye, significant refractive error (myopia ≤−0.75 diopters (D), hyperopia ≥2.00 D, or astigmatism ≥1.00 D) and visual acuity improving to >6/12 in both eyes with refraction.

^†^15 children who were lost to follow up had missing values for this variable.

^‡^Glasses wear at endline was defined as wearing glasses during an unannounced examination.

Comparing each group with the Control Group (cluster effect was adjusted):

^§^ P < 0.05

Significant predictors of having purchased spectacles in multiple regression models included: membership in the Free Glasses group (significantly reduced purchase: Relative Risk [RR] = 0.54, 95% Confidence Interval [95% CI] 0.34, 0.87, P = 0.01); Yunnan residence (significantly increased purchase: RR = 1.67, 95% CI 1.13, 2.45, P = 0.010); and studying for more than two hours per day (significantly increased purchase: RR = 1.65, 95% CI 1.07, 2.53, P = 0.02). Membership in the two Free + Upgrade groups, wearing glasses at baseline, uncorrected visual acuity and family wealth were unassociated with glasses purchase in the multivariate model. (Table 3).

**Table 3:** Effect of randomization group and other potential determinants on purchase of glasses prior to endline (main study outcome) adjusting for cluster effects within school.

Variable	Simple Regression Analysis		Multiple Regression Analysis (N = 882)†	
	Relative Risk (95% CI)	*P value*	Relative Risk (95% CI)	*P value*
Control Group as reference				
**Free Group**	**0.54 (0.34, 0.86)**	**0.010**	0.54 (0.34, 0.87)	**0.01**
Free + $15 Upgrade glasses group	0.91 (0.57, 1.45)	0.68	0.90 (0.56, 1.43)	0.65
Free + $30 Upgrade glasses group	0.90 (0.55, 1.48)	0.68	0.91 (0.59, 1.42)	0.69
Age (years)	0.92 (0.79, 1.07)	0.26		
Male sex	0.82 (0.62, 1.08)	0.15	0.84 (0.64, 1.10)	0.21
Wearing glasses at baseline*	**0.58 (0.35, 0.98)**	**0.04**	0.63 (0.38, 1.06)	0.09
**Yunnan residence**	**1.63 (1.07, 2.49)**	**0.02**	**1.67 (1.13, 2.45)**	**0.010**
Uncorrected visual acuity in eye with better vision	0.72 (0.40, 1.31)	0.28		
Only child in family	1.01 (0.69, 1.47)	0.97		
One or both parents with ≥12 years of education	1.19 (0.86, 1.64)	0.30		
Both parents away from the home the majority of time	1.02 (0.72, 1.44)	0.91		
At least one parent wears glasses	1.03 (0.74, 1.42)	0.88		
Learning time each day after school				
< 0.5 hr (as reference)				
0.5–1 hr	0.92 (0.68, 1.24)	0.60	0.93 (0.68, 1.27)	0.65
1–2 hrs	1.10 (0.74, 1.64)	0.63	1.19 (0.81, 1.75)	0.38
>2 hrs	1.51 (0.94, 2.42)	0.09	**1.65 (1.07, 2.53)**	**0.02**
Family wealth: (Bottom tercile as reference)				
Bottom tercile				
Middle tercile	1.15 (0.83, 1.59)	0.40		
Top tercile	1.19 (0.84, 1.68)	0.33		

*Defined as having glasses to hand at baseline, having previously been told to bring them to school.

^†^Variables with P-value< = 0.20 in simple regression models were included in multiple regression model analysis.

Significant predictors of observed glasses wear at endline in multiple models included wearing glasses at baseline (increased wear: RR = 3.01, 95% CI 2.32, 3.89, P < 0.001) and uncorrected visual acuity (children with better visual acuity were less likely to wear glasses: RR = 0.24, 95% CI 0.11, 0.50, P < 0.001). Parental education and glasses wear, both parents being away from home the majority of the time, ownership of glasses by friends, and family wealth were unassociated with glasses wear in the multivariate models, though several of these were significantly associated in univariate analyses. (Table 4) Sensitivity analyses were performed excluding children undergoing non-cyclopegic refraction with adjustment by the refractionist, and our main study findings were unchanged (data not shown).

**Table 4:** Effect of randomization group and other potential determinants on observed wear of glasses at endline examination among children needing glasses (Secondary study outcome), adjusting for cluster effects within school.

Variable	Simple Regression Analysis		Multiple Regression Analysis (N = 882)†	
	Relative Risk (95% CI)	*P Value*	Relative Risk (95% CI)	*P Value*
Individual level Control as reference				
Free group	1.57 (0.97, 2.56)	0.07	1.41 (0.93, 2.15)	0.11
Free + $15 Upgrade glasses group	1.16 (0.69, 1.94)	0.57	1.21 (0.80, 1.85)	0.37
Free + $30 Upgrade glasses group	0.88 (0.46, 1.66)	0.68	0.95 (0.51, 1.76)	0.86
Age (years)	0.89 (0.79, 1.01)	0.06	0.92 (0.81, 1.05)	0.21
Male sex	0.92 (0.71, 1.19)	0.53		
Wearing glasses at baseline*	**4.62 (3.65, 5.86)**	**<0.001**	**3.01 (2.32, 3.89)**	**<0.001**
Yunnan residence	1.18 (0.76, 1.83)	0.47		
**Uncorrected visual acuity in eye with better vision**	**0.07 (0.03, 0.16)**	**<0.001**	**0.24 (0.11, 0.50)**	**<0.001**
Only child in family	1.29 (0.96, 1.74)	0.10		
**One or both parents with ≥12 years of education**	**1.33 (1.04, 1.69)**	**0.02**	1.13 (0.90, 1.42)	0.28
**Both parents away from the home the majority of time**	**0.66 (0.45, 0.97)**	**0.04**	0.70 (0.48, 1.01)	0.06
**At least one parent wears glasses**	**1.70 (1.33, 2.18)**	**<0.001**	1.16(0.92, 1.47)	0.21
Learning time each day after school				
<0.5 hr as reference				
0.5–1 hr	1.14 (0.85, 1.54)	0.38	1.03 (0.79, 1.34)	0.84
1–2 hrs	1.23 (0.84, 1.81)	0.28	0.97 (0.68, 1.37)	0.85
> 2 hrs	**1.64 (1.18, 2.27)**	**0.003**	1.31 (0.92, 1.87)	0.13
**Do you have friends wearing glasses?**				
No (as reference)				
Yes	**1.57 (1.15, 2.15)**	**0.004**	1.09 (0.84, 1.43)	0.51
I don’t know	1.11 (0.66, 1.86)	0.70	1.12 (0.70, 1.79)	0.64
Family wealth: (Bottom tercile as reference)				
Bottom tercile				
Middle tercile	1.40 (0.99, 1.98)	0.06	1.03 (0.76, 1.38)	0.86
**Top tercile**	**1.46 (1.09, 1.96)**	**0.01**	1.03 (0.78, 1.37)	0.82

*Defined as having glasses to hand at baseline, having previously been told to bring them to school.

^†^Variables with P-value< = 0.20 in simple regression models were included in multiple regression model analysis.

## Discussion

As hypothesized, providing free glasses in this trial did significantly reduce purchase in the Free Glasses group (12.7% rate of purchase versus 23.6% among Controls who received no free glasses), but offering the opportunity to purchase upgrade glasses negated this effect (21.3% and 20.9% rates of purchase in the two Upgrade groups). At this price range of US$15–30 (chosen as the interquartile range of price paid for glasses in our previous studies) [9], demand for glasses was relatively inelastic.

The results of this trial have important implications for spectacle programs: evidence suggests that free glasses can significantly increase rate of wear [9, 26], but without the potential for glasses sales, these programs are likely to be unsustainable. This trial suggests that offering free glasses need not undercut sales. In China, for example, distribution of free spectacles by the government might go forward without undermining the profitability of spectacle sales by local hospitals, needed to support school screenings to identify children with refractive error. It is particularly encouraging in this context that rates of purchase (40%) were actually significantly higher in Yunnan, one of China’s poorest provinces, when compared to far richer Guangdong. Further research is needed to calibrate cost versus demand curves in order to maximize income without reducing adherence.

Observed spectacle wear rates in this setting were low (23.2% at endline even with provision of free spectacles). This is comparable with our previous results using the identical protocol, among similar-aged children in Shaanxi (19.5%) and Gansu (16.8%) [9]. In part, this is due to our choice of a conservative definition of spectacle wear, only counting those children observed to have glasses on their face at the time of an un-announced examination. This almost certainly under-estimated true rates of wear among children, but was felt to be more reliable than asking children about their own glasses use, and more practical than asking teachers to record wear over time. The principal goal of the current study was to better understand factors impacting on spectacle purchase and not wear, particularly the use of upgrade glasses. Our recent trial in urban migrant communities in eastern China [12] has already demonstrated that teacher incentives offer an effective means of optimizing children’s long-term spectacle use in the classroom, achieving wear in nearly three-quarters of children over the course of a school year. Had such interventions been used in the current study, it is likely that wear rates would have been higher.

Strengths of this study include its randomized controlled design, population-based sampling in the selected areas, high rates of follow-up and of accurate implementation of interventions, programmatically relevant choice of county hospital as distribution points for spectacles and the inclusion of both rich and poor provinces. Weaknesses include having chosen only counties in two provinces where local hospitals interested to take part in the study could be found, which may limit generalizability to other regions of China. Further, we did not attempt in the current paper to calculate the proportion of program costs which sale of upgrade spectacles might support in these settings, which may be valuable to attempt in the future. A proportion of children underwent non-cyclopegic refraction, due to their parents failing to give permission for cycloplegia. These children had their automated refraction values adjusted by experienced refractionists using targets at a distance of 4 meters in order to remove the effects of accommodation. An alternative might have been to use retinoscopy with a distant target to reduce the effects of instrument accommodation, but this technique is not widely used in rural China and was not practical under the circumstances. Sensitivity analyses showed that the impact on our conclusions of using non-cycloplegic refraction on some children appears to have been minimal. Finally, the study was not designed to explore in depth the economic models employed by optical shops in these areas.

We searched PubMed in April 2014, using the terms “refractive error” and “myopia,” cross-indexed with “glasses” and “spectacles,” and “sale, “purchase” and “distribution” for articles published in any language since Jan 1, 1970. We found no previous randomized trials designed to examine the effect of providing free glasses on families’ purchase of children’s spectacles. Previous trials in China [9], Tanzania [13] and India [27] have shown that providing free spectacles increases rates of wear significantly, when compared to providing prescriptions and depending on families to purchase glasses themselves. Other hybrid pricing models have been examined in non-trial settings, including a study in Timor-Leste [28] demonstrating that cross-subsidization could cover most costs of a rural spectacle program.

## Conclusions

The PRICE model of “free glasses for all with an optional upgrade” may be an important one for China and other countries with a high prevalence of refractive error and a growing middle class, where spectacle sales may potentially be important to sustain programs of distribution.

## Supporting information

S1 File Baseline student questionnaire_English.doc. English version of baseline student questionnaire.
(DOC)

S2 File Baseline students questionnaire_Chinese.pdf. Chinese version of baseline student questionnaire.
(PDF)

S3 File PRICE endline students questionnaire_English.pdf. English version of endline student questionnaire.
(PDF)

S4 File PRICE endline students questionnaire_Chinese.pdf. Chinese version of endline student questionnaire.
(PDF)
