# Supplementary material for: Cluster-randomized controlled trial of the effects of free glasses on purchase of children's glasses in China: The PRICE (Potentiating Rural Investment in Children's Eyecare) study
Source: PLoS One. 2017 Nov 21;12(11):e0187808. doi: 10.1371/journal.pone.0187808 (PMC5697864; doi:10.1371/journal.pone.0187808)
Supplement: S1 File — English version of baseline student questionnaire. (DOC) [file pone.0187808.s001.doc]

**：**

**Baseline Student Form**

| **_______________District** | **_____________Street/Township** | |
| --- | --- | --- |
| **School:**  **Name:**  **Your seat: Row Line** | **________________Year**  **Ethnic group：** | **_____________class** |

# Basic Information

| **Questions** |  | **Answers** | | | | | | | | | | | |  |
| --- | --- | --- | --- | --- | --- | --- | --- | --- | --- | --- | --- | --- | --- | --- |
| 1. What is your gender? | 1=Male 2=Female |  | | | | | | | | | | | |  |
| 1. What is your age? | Year |  | | | | | | | | | | | |  |
| 1. How many siblings (same parents) do you have? (Please fill in ‘0’ if you do not have sibling) |  | Elder brother： | | | | | | Elder  sister： | | | | | |  |
|  |  | Younger brother： | | | | | | Younger sister： | | | | | |  |
| 1. If you have siblings (same parents), how many of them have glasses? (Please fill in ‘0’ if none of them have glasses) |  |  | | | | | | | | | | | |  |
| 1. Does your father wear glasses (Except Sunglasses)? | 1=Yes 2=No |  | | | | | | | | | | | |  |
| 1. What is your father’s education level? | 1=No schooling 2=Primary school  3=Junior high school 4=High school/professional high school  5=Professional college 6=Higher than college |  | | | | | | | | | | | |  |
| 1. Did your father live at home in most of last term? | 1=Yes 2=No |  | | | | | | | | | | | |  |
| 1. Does your mother wear glasses (Except Sunglasses)? | 1=Yes 2=No |  | | | | | | | | | | | |  |
| 1. What is your mother’s education level? | 1=No schooling 2=Primary school  3=Junior high school 4=High school/professional high school  5=Professional college 6=Higher than college |  | | | | | | | | | | | |  |
| 1. Did your mother live at home in most of last term? | 1=Yes 2=No |  | | | | | | | | | | | |  |
| 1. What is the phone number of your home? | You can fill in multiple number. Please separate them by coma. |  |  |  |  |  |  | |  |  |  |  |  | |
|  |  |  |  |  |  |  |  |  |  |  |  |  |  |  |

# Vision Status

| **Question** | **Alternatives** | **Answer** |
| --- | --- | --- |
| 1. How long do you read and write your homework after school every day? | 1=Not read or write；  2=Less than half hour;  3=Half hour to one hour;  4=One hour to two hours;  5=Two hours to three hours;  6=More than 3 hours |  |
| 1. How much proportion of tests questions is written on the blackboard in your class? | 1= All; 2= Most; 3= About half;  4= less; 5= None |  |
| 1. Can you clearly read the words on the blackboard in your seat? | 1= Can see clearly; 2= Can't see clearly |  |
| 1. Do you think you are near sighted? | 1=Yes 2=No |  |
| 1. Do you think it is ugly to wear glasses? | 1=It is ugly;  2=It is a bit ugly;  3=It is ordinary;  4=It is not ugly;  5=It is nice |  |
| 1. Are there any classmates in your class who are being bullied by other classmates because of wearing glasses? | 1=Yes； 2=No |  |
| 1. Do any of your good friends have glasses? | 1=Yes；  2=No；  3=I do not know； |  |
| 1. Do you have glasses now? | 1=No >>>>>>>>>>>>> (**directly into the third part**);  2=Yes, but I didn't bring it to school today;  3=Yes, I took them to school today |  |

**II. Vision Status (Continue, Only students who choose "2" or "3" (with glasses) for question 19 need to answer question 20-24)**

| **Question** | **Alternatives** | **Answer** |
| --- | --- | --- |
| 1. Do you usually wear glasses? | 1= Basically don't wear;  2= Wear while learning;  3= Always wear; |  |
| 1. If you don't always wear glasses, why?   (Choose only one answer) | 0= I always wear glasses;  1= I worry about my vision will become worse and worse if I wear glasses;  2= I am afraid of being mocked or bullied if I wear glasses.  3=I can still see without glasses now. I will wear glasses when I cannot see clearly;  4=I feel it is inconvenient to wear glasses, especially when doing exercise.  5=It is uncomfortable to wear glasses, and I will feel dizzy, nose pressure, etc.;  6=Other reasons, please tell us__ |  |
| 1. What's the attitude of your parents as to your wearing glasses? | 1= They support it;  2= They doesn't care;  3= They against it |  |
| 1. Where do you fit your glasses? | 1=Hospital 2=Optical shop  3=Other place, please tell us__ |  |
| 1. How much did you pay for your glasses? | 1= Under 100 RMB;  2=100 to 199 RMB;  3=200 to 299 RMB;  4=300 to 399 RMB;  5=400 to 499 RMB;  6=500 RMB and above |  |

1. **Usage of glasses (for the following statements, 1=Agree; 2=Disagree; 3=I don’t know)**

| 1. Eye exercise can cure Myopia | 1=Agree; 2=Disagree;3=I don’t know |  |
| --- | --- | --- |
| 1. Wearing glasses can cure Myopia | 1=Agree; 2=Disagree;3=I don’t know |  |
| 1. For those with **lower grades** and **myopia**, wearing glasses will **improve**their grades | 1=Agree; 2=Disagree;3=I don’t know |  |
| 1. For those who **cannot** see the blackboard clearly, wearing glasses make them see the blackboard **more clearly** | 1=Agree; 2=Disagree;3=I don’t know |  |
| 1. Wearing glasses will make your vision worse | 1=Agree; 2=Disagree;3=I don’t know |  |

**IV. Family ownership of assets**

| Asset class | 1=Yes；2=No | Asset class | 1=Yes；2=No |
| --- | --- | --- | --- |
| 1. Car |  | 1. Camera |  |
| 1. Truck |  | 1. Washing machine |  |
| 1. Motorcycles or electro-mobile |  | 1. Air conditioner |  |
| 1. Tractor |  | 1. Water heater |  |
| 1. Large agricultural implements |  | 1. Gas / liquid gas stove |  |
| 1. Computer |  | 1. Kitchen ventilator |  |
| 1. Computer Internet |  | 1. Refrigerator or freezer |  |
| 1. Television |  | 1. Flush toilet |  |

**Vision screening form**

**Part 1: Vision Check**

| **Uncorrected Vision** | |
| --- | --- |
| 1. OD | 1. OS |
| □6/3  □6/3.8  □6/4.8  □6/6  □6/7.5  □6/9.5  □6/12  □6/15  □6/19  □6/24  □6/30  □6/38  □6/48  □6/60  □6/76  □6/96  □6/120  □6/152  □6/192  □6/240  □< 6/240 | □6/3  □6/3.8  □6/4.8  □6/6  □6/7.5  □6/9.5  □6/12  □6/15  □6/19  □6/24  □6/30  □6/38  □6/48  □6/60  □6/76  □6/96  □6/120  □6/152  □6/192  □6/240  □< 6/240 |

1. Do you have glasses?

| No，I do not have. Go to Part 2.  Yes, I have. | | |  |
| --- | --- | --- | --- |
| If you have glasses, please check the Corrected Vision. | | |  |
| **Corrected Vision** | |  | |
| 1. OD | 1. OS |  | |
| □6/3  □6/3.8  □6/4.8  □6/6  □6/7.5  □6/9.5  □6/12  □6/15  □6/19  □6/24  □6/30  □6/38  □6/48  □6/60  □6/76  □6/96  □6/120  □6/152  □6/192  □6/240  □< 6/240 | □6/3  □6/3.8  □6/4.8  □6/6  □6/7.5  □6/9.5  □6/12  □6/15  □6/19  □6/24  □6/30  □6/38  □6/48  □6/60  □6/76  □6/96  □6/120  □6/152  □6/192  □6/240  □< 6/240 |  | |

# Part 2: Inclusion Criteria

1. Is this student selected?

**Yes,** uncorrected (without glasses) visual acuity of ≤6/12 in either eye

**No**
