# Supplementary material for: Cluster-randomized controlled trial of the effects of free glasses on purchase of children's glasses in China: The PRICE (Potentiating Rural Investment in Children's Eyecare) study
Source: PLoS One. 2017 Nov 21;12(11):e0187808. doi: 10.1371/journal.pone.0187808 (PMC5697864; doi:10.1371/journal.pone.0187808)
Supplement: S2 File — Chinese version of baseline student questionnaire. (PDF) [file pone.0187808.s002.pdf]

调查员编码： _____________　　　　　　　　　　　　　　　　　　填表日期:________________

问卷编码：（«学校编码»）　　　　　«学生编码»）

小学生基本情况调查

同学你好：

今天让大家来填写一份问卷。我们会为你的答案保密，你的答案不会告诉调查组以外的任何人。这些问题也并没有标准答案， 希望你不要有任何顾虑，跟随我们准确、如实、完整地填写这份调查表。如果你在填这份问卷时有任何问题，请随时举手问我们。衷心感谢你对本次调研工作的积极支持与配合！祝你身体健康！学习进步！

农村教育行动计划课题组

（«省»）省（«县»）县(区)_________镇(乡)______________小学___年级______班

姓名：_________________

请填写你座位所在的排和列(最靠近讲台的一排为第一排;最靠近门的一边为第一列)：

____________排____________列

你的民族？（请在下表中你所在的民族后打钩）

汉族		傣族		彝族		白族	
拉祜族族		景颇族		佤族		哈尼族	
其他，请注明							

答题须知：

1. 每个表格中有三列：“问题”、“单位/选项”和“答案”。如果是选择题，请将您认为最符合您情况和想法的选项的**代码**填在选项后面“答案”列的空格里。如果是问答题，请把您的答案用文字填在选项后面“答案”列的空格里。
2. 如无特别说明，本问卷中所有的问题都只选一个答案。
3. 数字要写阿拉伯数字，比如1、2、3。
4. 如果选择题选项中有“其他，请说明____”这样的字样，您也选了这项，请在“答案”列中填上这项的代码，并在“答案”列同时填上需要说明的内容。
5. **不能用铅笔**填写。

一、基本信息

问题	选项	答案
1.你的性别？	1=男；　2=女	
2.你几岁了？	周岁	
3.你有几个亲兄弟姐妹？	人（如果没有请填“0”）	
4.如果**有亲兄弟姐妹**，有几个戴眼镜？	人（如果没有请填“0”）	
5.你爸爸戴眼镜吗？（**太阳镜**除外）	1=戴；　　2=不戴	
6.你爸爸的文化程度？	1=没上过学；2=小学；<br>3=初中；　4=高中或中专；<br>5=大专；　6=大学及以上	
7.上个学期，你爸爸大部分时间在家住吗？	1=是；<br>2=否；	
8.你妈妈戴眼镜吗？（**太阳镜**除外）	1=戴；　　2=不戴	
9.你妈妈的文化程度？	1=没上过学；2=小学；<br>3=初中；　4=高中或中专；<br>5=大专；　6=大学及以上	
10.上个学期，你妈妈大部分时间在家住吗？	1=是；<br>2=否；	
11.你家的电话号码是多少？	可填写多个手机或座机号码，一行写一个号码	

二、视力现状

问题	选项	答案
12.你每天放学后看书、写作业的时间？	1=不看书或写作业；　2=半小时以内；<br>3=半小时到一小时；　4＝一小时至两小时；<br>5=二小时至三小时；　6=三小时以上	
13.老师平时上课出的测试题，有多大比例是写在黑板上的？	1=全部；　2=大部分；　3=一半左右；<br>4＝很少；　5=没有	
14.你平时在座位上能不能看得清楚黑板上的字？	1=看得清楚；　　2=看不清楚	
15.你觉得你近视吗？	1=是；　　2=否	
16.你觉得戴眼镜难看吗？	1=难看；　2=有点难看；　3=一般；<br>4=不难看；　5=很好看	
17.你们班有同学因为戴眼镜被别的同学笑话欺负吗？	1=有；　　2=没有	
18.你的好朋友当中，有人有眼镜吗？	1=有；　2=没有；　3=不知道	
19.你现在有眼镜吗？	1=没有>>>>>>>>>>>>>（**直接做第三大题**）；<br>2=有，但今天没有带到学校；<br>3=有，今天带到学校了	

二、视力现状（续，仅第 19 题选择“2”或者“3”（有眼镜）的同学需要回答第 20-24 题）

问题	选项	答案
20. 你平时戴眼镜吗？	1=基本不戴； 2=学习的时候戴；<br>3=经常戴	
21. 如果你不是经常戴眼镜，你自己主要是怎么考虑的？<br>**（只能选一个答案）**	0=我经常戴眼镜；<br>1=担心戴了眼镜后视力越来越差；<br>2=担心戴了眼镜被别人笑话；<br>3=不戴眼镜也勉强看得见，等以后看不清楚了再戴；<br>4=觉得戴着眼镜行动(或体育活动)不方便；<br>5=戴眼镜不舒服，会觉得头晕、鼻梁受压等；<br>6=其他，请注明	
22. 你的家长对你戴眼镜是什么态度？	1=支持； 2=无所谓； 3=反对	
23. 你的眼镜是在哪里配的？	1=医院； 2=眼镜店； 3=其他，请说明	
24. 你配眼镜花了多少钱？<br>Text	1=100 元以下；<br>2=100 元到 199 元；<br>3=200 元到 299 元；<br>4=300 元到 399 元；<br>5=400 元到 499 元；<br>6=500 元及以上	

三、眼镜使用问题 （对于以下说法，同意写 1；不同意写 2；不知道写 3）

25. 眼保健操能矫正近视	1=同意；2 不同意；3=不知道	
26. 戴眼镜能矫正近视	1=同意；2 不同意；3=不知道	
27. 对于成绩<u>比较差</u>，同时又<u>近视</u>的学生，戴眼镜会<u>提高</u>学习成绩	1=同意；2 不同意；3=不知道	
28. 对于<u>看不清楚</u>黑板的学生，戴眼镜会让他们看得<u>更清楚</u>	1=同意；2 不同意；3=不知道	
29. 戴眼镜会导致视力越来越差	1=同意；2 不同意；3=不知道	

四、家庭资产拥有情况

资产类别	1=有；2=没有	资产类别	1=有；2=没有
1. 小汽车		2. 照相机	
3. 货车		4. 洗衣机	
5. 摩托车或电动车		6. 空调	
7. 拖拉机		8. 热水器	
9. 大型农机具		10. 煤气/液化气炉具	
11. 电脑		12. 抽油烟机	
13. 拉网线（电脑上网）		14. 电冰箱或冰柜	
15. 电视机		16. 抽水马桶	

谢谢，问卷结束！

学生视力筛查表

第一部分：视力筛查

1. 检查日期：2014 年___月_____ 日

裸眼视力	
2. 右眼	3. 左眼
☐6/3	☐6/3
☐6/3.8	☐6/3.8
☐6/4.8	☐6/4.8
☐6/6	☐6/6
☐6/7.5	☐6/7.5
☐6/9.5	☐6/9.5
☐6/12	☐6/12
☐6/15	☐6/15
☐6/19	☐6/19
☐6/24	☐6/24
☐6/30	☐6/30
☐6/38	☐6/38
☐6/48	☐6/48
☐6/60	☐6/60
☐6/76	☐6/76
☐6/96	☐6/96
☐6/120	☐6/120
☐6/152	☐6/152
☐6/192	☐6/192
☐6/240	☐6/240
☐< 6/240	☐< 6/240

4. 现在是否有眼镜？

☐ 没有，转到第二部分

☐ 有，没带来，转到第二部分

☐ 有，带来了

如果有并且带来了，测试戴上现有眼镜视力：

戴镜视力	
5. 右眼	6. 左眼
☐6/3	☐6/3
☐6/3.8	☐6/3.8
☐6/4.8	☐6/4.8
☐6/6	☐6/6
☐6/7.5	☐6/7.5
☐6/9.5	☐6/9.5
☐6/12	☐6/12
☐6/15	☐6/15
☐6/19	☐6/19
☐6/24	☐6/24
☐6/30	☐6/30
☐6/38	☐6/38
☐6/48	☐6/48
☐6/60	☐6/60
☐6/76	☐6/76
☐6/96	☐6/96
☐6/120	☐6/120
☐6/152	☐6/152
☐6/192	☐6/192
☐6/240	☐6/240
☐< 6/240	☐< 6/240

第二部分：入选标准

7. 是否入选

是，任一眼裸眼视力≤ 6/12(0.5) ☐

否 ☐
