# Supplementary material for: Cluster-randomized controlled trial of the effects of free glasses on purchase of children's glasses in China: The PRICE (Potentiating Rural Investment in Children's Eyecare) study
Source: PLoS One. 2017 Nov 21;12(11):e0187808. doi: 10.1371/journal.pone.0187808 (PMC5697864; doi:10.1371/journal.pone.0187808)
Supplement: S3 File — English version of endline student questionnaire. (PDF) [file pone.0187808.s003.pdf]

Investigator ID: ____________ Date: ______________

Participant unique ID ________________

Potentiating Rural Investment in Children's Eye care (PRICE) End Line Survey

Vision and Eye Glasses Wearing of Primary School Students

Dear students:

We are now conducting the end line survey of our PRICE study. Can you help us fill in a questionnaire, which can help us know about your vision and eye glasses wearing habits? There are NO standard answers for these questions and your answers are SAFE with us. We will NOT release your answers to anyone else outside the research team. We hope you can answer these questions according to your own situation and let us know about your thoughts. If you have any question, please feel free to raise your hand and ask our onsite investigators.

Thank you very much for your participation in our PRICE project and your support for our investigation!

With the best wishes from the entire research team!

Province __________ City / County / District ___________ Town(Village) __________

School ____________ Grade ______ Class ______ Name __________

Instructions:

1. There are three columns in the questionnaire: "Questions", "Choices/Options" and "Answers". Please fill in the code that fits your situation the best in the "Answers" Section. If you choose "The other reason, please tell us_______", please fill in both the code and the text in the "Answers" Section.
2. Please just choose <u>ONLY ONE</u> answer for all the questions, unless otherwise specified.
3. Please use the Arabic numerals, such as 1, 2 and 3.
4. Please do <u>NOT</u> use pencil to fill in the questionnaire.

Part I: About your glasses

Questions	Choices / Options	Answers
1. Date of birth (DOB)	__________Year_________Month_______Day	
2. Do you have glasses?	1=No >>> （**Direct to the fourth part**）；<br>2=Yes	
3. Do you think the glasses are helpful for your study and life?	1=Very helpful； 2=More or less；<br>3=Not really； 4=Not at all；	
4. What do you think of your look with glasses?	1=Pretty good； 2=Just so so；<br>3=Not that good； 4=I do not care about it.	
5.What is the attitude of your parents as to your wearing glasses?	1=They support it； 2=They do not care；<br>3=They are against it.	
6. How often do you wear your glasses?	1=Basically never； 2=Only when studying；<br>3=Quite often	
7. If you basically never wear or only wear your glasses when studying, what is your main concern?<br><br>(**Only one choice please)**	0=I often wear my glasses (If you chose "3" in Q6, please choose "0" for this question.)<br>1=I worry about my vision will become worse and worse if I wear glasses.<br>2=I worry about being mocked and bullied if I wear glasses.<br>3=I can still see without glasses now, I will wear glasses when I cannot see clearly.<br>4= I feel it is inconvenient to wear glasses, especially when doing exercise.<br>5=It is not very comfortable to wear glasses (e.g. dizziness).<br>6=I do not like my look with glasses.<br>7=Other reasons, please tell us__________	
8. When did you have your current glasses?	1=After the vision screening of the PRICE project in last semester.<br>2=I had my glasses before the vision screening. I got the glasses in ______Year ___Month.	
9. Do you have glasses from our project?	0=No；>>> **Direct to Part Ⅲ**<br>1=Yes, I got the free glasses; >>> To Part Ⅱ<br>2=Yes, with 100 RMB to get upgrade glasses A; >>> To Part Ⅱ<br>3=Yes, with 200 RMB to get upgrade glasses B; >>> To Part Ⅱ	

Part Ⅱ: Evaluation of the project glasses (For those who chose 1, 2 or 3 in Q9), please answer Q10 – Q12.

10. If you choose the upgrade glasses (A or B), what is your main concern ?<br><br>(**Only one choice please)**	0=I chose the free project glasses. (If you choose "1" in Q9, please choose "0" in this question.)<br>1=I worried about the quality of the free glasses, and assumed the quality for the upgrade glasses may be better	

	with extra pay.<br>2=Upgrade glasses look better.<br>3=My parents chose them for me.<br>4=Other reasons, please tell us__________	
11. What do you think of the styles of our project glasses?	1=Pretty good, I like it. 2=Just so so；<br>3=Bad； 4=I don't care, since it is for free.	
12. Do you see clearly with our project glasses?	1=Yes, quite clearly； 2=Fine；<br>3=Not very clearly；	

Part Ⅲ: The price of your own glasses (ONLY for students with non-project glasses)

13. If your glasses are purchased outside our project, how much are your glasses?<br>**<u>(Students who chose the project glasses, please skip this question)</u>**	1=Less than 100 RMB<br>2=100 to 199 RMB；<br>3=200 to 299 RMB；<br>4=300 to 399 RMB；<br>5=400 to 499 RMB；<br>6=500 RMB or more<br>If you can remember the exact price of your glasses, please fill in: ______RMB	

Part Ⅳ: For those chose "1" in Q2 and "0" in Q9

Questions	Choices / Options	Answers
14.Do you receive prescription or voucher from our project?	1=I received prescription. (Please answer Q15)<br>2=I received voucher. (Please answer Q16)<br>3=Neither； **>>> <u>End of the questionnaire</u>**	
15. If you received prescription, why didn't you purchase your glasses?<br><br>(**<u>Only one choice please)</u>**	1=I worry about my vision will become worse and worse if I wear glasses.<br>2=Almost no one I know wear glasses, I am afraid of being mocked or bullied if I wear glasses.<br>3=I can still see without glasses now. I will wear glasses when I cannot see clearly.<br>4=I feel it is inconvenient to wear glasses, especially when doing exercise.<br>5=My parents do not care about my vision.<br>6=My parents did not trust the accuracy of the prescription.<br>7=The glasses are too expensive for my family.<br>8=My parents/relatives did not have time to take me to buy glasses (or inconvenient for transportations).<br>9=Other reasons, please tell us_____________	
16. If you received voucher, why didn't you get our project glasses?<br><br>(**<u>Only one choice please)</u>**	0=I already have my glasses, and I do not need a new pair for now.<br>1= I worry about my vision will become worse and worse if I wear glasses.<br>2= Almost no one I know wear glasses, I am afraid of being mocked or bullied if I wear glasses.<br>3= I can still see without glasses now. I will wear glasses when I cannot see clearly.<br>4= I feel it is inconvenient to wear glasses,	

	especially when doing exercise.<br>5=My parents do not care about my vision.<br>6=My parents did not believe we can get a free pair of glasses.<br>7= My parents/relatives did not have time to take me to buy glasses (or inconvenient for transportations).<br>8=We were given a very urgent notice and a very short period to choose glasses.<br>9=Other reasons, please tell us_____________	

This is the end of the questionnaire.

Vision Re-check Form

Part 1: Vision Check

3. Do you have glasses?
☐ No. End of survey.
☐ Yes. But I do not bring with me. End of survey.
☐ Yes, I do. Check corrected vision with glasses.

My glasses are:
☐ Project glasses
☐ Non-project glasses

Uncorrected Vision	
**1.** OD	**2.** OS
□6/3	□6/3
□6/3.8	□6/3.8
□6/4.8	□6/4.8
□6/6	□6/6
□6/7.5	□6/7.5
□6/9.5	□6/9.5
□6/12	□6/12
□6/15	□6/15
□6/19	□6/19
□6/24	□6/24
□6/30	□6/30
□6/38	□6/38
□6/48	□6/48
□6/60	□6/60
□6/76	□6/76
□6/96	□6/96
□6/120	□6/120
□6/152	□6/152
□6/192	□6/192
□6/240	□6/240
□< 6/240	□< 6/240

Corrected Vision	
**4.** OD	**5.** OS
□6/3	□6/3
□6/3.8	□6/3.8
□6/4.8	□6/4.8
□6/6	□6/6
□6/7.5	□6/7.5
□6/9.5	□6/9.5
□6/12	□6/12
□6/15	□6/15
□6/19	□6/19
□6/24	□6/24
□6/30	□6/30
□6/38	□6/38
□6/48	□6/48
□6/60	□6/60
□6/76	□6/76
□6/96	□6/96
□6/120	□6/120
□6/152	□6/152
□6/192	□6/192
□6/240	□6/240
□< 6/240	□< 6/240

Part 2: Glasses quality check 6.Does the student selected for quality check?
☐ No, end of the survey. ☐ Yes, continue the following part.

Refraction:

	SPH	CYL	AXIS	BCV
OD	**7.**	**8.**	**9.**	**10.**
OS	**11.**	**12.**	**13.**	**14.**

Prescription:

	SPH	CYL	AXIS	BCV	PD
OD	**15.**	**16.**	**17.**	**18.**	23.
OS	**19.**	**20.**	**21.**	**22.**	

Lensometer:

	SPH	CYL	AXIS	BCV	PD
OD	**24.**	**25.**	**26.**	**27.**	32.
OS	**28.**	**29.**	**30.**	**31.**	

33. How is the difference between the strength of project glasses detected by the lesometer and the prescription?
☐ The margin error is acceptable. ☐ The difference is out of the national standards.
