# Supplementary material for: Cluster-randomized controlled trial of the effects of free glasses on purchase of children's glasses in China: The PRICE (Potentiating Rural Investment in Children's Eyecare) study
Source: PLoS One. 2017 Nov 21;12(11):e0187808. doi: 10.1371/journal.pone.0187808 (PMC5697864; doi:10.1371/journal.pone.0187808)
Supplement: S4 File — Chinese version of endline student questionnaire. (PDF) [file pone.0187808.s004.pdf]

调查员编码：________　　　　　　　　　　　　　　　　　　　　　填表日期: _______

问卷编码 ID：（）

加强农村儿童眼保健项目

小学生配戴眼镜情况调查

亲爱的同学：

你好！今天请你帮我们填写一份问卷。这份问卷能帮助我们了解你的视力和眼镜的情况。我们会为你的答案保密，不会将你的答案告诉调查组以外的任何人。我们这些问题也没有标准答案，希望你不要有任何顾虑，准确、如实、完整地告诉我们你的想法即可。如果你在填写问卷过程中有任何问题，请随时举手示意我们的工作人员。衷心感谢你对我们调研工作的支持！祝你身体健康！学习进步！

农村教育行动计划课题组

___省__市 / 县 / 区___镇(乡)_小学___年级__班

姓名：_

答题须知：

1. 每个表格有三列："问题"、"选项"和"答案"。如果是选择题，请将你认为最符合你情况或想法的选项**代码**填写在"答案"列的空格里。如果你选择了"其他原因，请告诉我们____"这样的选项，请在"答案"列中填上这个选项的代码，并在横线上填写文字内容。
2. 如无特别说明，本问卷中所有的问题都只选一个答案。
3. 数字请用阿拉伯数字，比如1、2、3。
4. **请不要用铅笔**填写。

第一部分：关于你的眼镜

问题	选项	答案
1. 请问你的出生日期是？（请将答案写在横线上）	出生日期：___________年__________月________日	
2. 你有眼镜吗？	1=没有>>>>>>>>>>>>>>（**请直接做第四部分**）；<br>2=有	
3. 你认为眼镜对学习和生活有帮助吗？	1=非常有帮助； 2=有一点帮助；<br>3=眼镜对我来说可有可无； 4=毫无帮助；	
4. 你觉得自己戴眼镜好看吗？	1=好看； 2=一般；<br>3=不好看； 4=无所谓，我不关心	
5. 家长对你戴眼镜是什么态度？	1=支持； 2=无所谓； 3=反对	
6. 你是否经常戴眼镜？	1=基本不戴； 2=学习的时候戴； 3=经常戴	
7. 如果你**基本不戴或者仅学习的时候戴眼镜**，你自己**主要**是怎么考虑的？<br>（**单选**，答案序号写于答案列，若选 7 请将具体原因填在横线上）	0=我经常戴眼镜（**如果第 6 题选择"3"，此题请选"0"**）<br>1=担心戴了眼镜后视力越来越差；<br>2=担心戴了眼镜被别人笑话或欺负；<br>3=不戴眼镜也勉强看得见，等以后看不清楚了再戴;<br>4=觉得戴着眼镜行动(或体育活动)不方便；<br>5=戴眼镜不舒服，会觉得头晕、鼻梁受压等；<br>6=我不喜欢自己戴眼镜的样子；<br>7=其他原因，请告诉我们___________	
8. 你现在戴的眼镜是什么时候配的？（答案序号写于答案列，并将具体时间填在横线上）	1=上学期项目组做视力检查后<br>2=我在上学期项目组做视力检查前就有眼镜了，<br>我在______年___月配眼镜	
9. 你的眼镜属于哪种情况？<br>（你有没有项目眼镜？）	0=不是项目组的眼镜；**》》》直接进入第三部分**<br>1=免费眼镜；》》第二部分<br>2=升级眼镜，交了 100 元；》》第二部分<br>3=升级眼镜，交了 200 元；》》第二部分	

第二部分：对项目眼镜的评价——选择项目眼镜的同学（第 9 题选择 1,2 或者 3）请回答 10-12 题：

10. 如果你的眼镜是项目组升级眼镜，当时买升级眼镜的**主要原因**是什么？<br>（**单选**，答案序号写于答案列，若选 4 请将具体原因填在横线上）	0=我选的是免费眼镜（**如果第 9 题选择"1"，此题请选"0"**）<br>1=担心免费眼镜不可靠，额外花钱的眼镜可能质量更好；<br>2=额外花钱的眼镜样子更好看；<br>3=父母帮我选择的；<br>4=其他原因，请列出：_________	
11. 你对你所挑选的项目组眼镜款式作何评价？	1=好看，我很喜欢； 2=一般；<br>3=不好看； 4=无所谓，反正是免费的	
12. 你使用项目组的眼镜看东西，感觉清晰吗？	1=清晰； 2=一般； 3=不清晰；	

第三部分：眼镜的价格——配戴非项目眼镜的同学请回答第 13 题

13. 如果你的眼镜不是项目组的眼镜，你的眼镜大概多少钱？<br>**<u>（选择项目眼镜的同学跳过此题）</u>**	1=100 元以下；<br>2=100 元到 199 元；<br>3=200 元到 299 元；<br>4=300 元到 399 元；<br>5=400 元到 499 元；<br>6=500 元及以上<br>如果你知道眼镜的具体价格，请在横线上填写具体价格：_____元	

第四部分：第 2 题选择 1（没有眼镜）/ 第 9 题选择"0"（配了非项目的眼镜）的同学回答第四部分

问题	选项	答案
14. 你是否收到项目组发的处方或者眼镜券？	1=我收到配镜处方；（请回答第 15 题）<br>2=我收到眼镜券；　（请回答第 16 题）<br>3=都没有；>>>>>>>>>>>**<u>问卷结束</u>**	
15. 如果收到处方，你为什么没去配眼镜？<br>（单选，答案序号写于答案列，若选 9 请将具体原因填在横线上）	1=担心戴了眼镜后视力越来越差；<br>2=我认识的人基本没有人戴眼镜，担心戴了眼镜被别人笑话或者被欺负；<br>3=不戴眼镜也勉强看得见，等以后看不清楚了再戴；<br>4=觉得戴着眼镜行动(或体育活动)不方便；<br>5=家长不重视我的视力情况；<br>6=爸爸、妈妈不相信处方单的准确性；<br>7=眼镜太贵，我家里买不起<br>8=爸爸、妈妈或亲朋好友不方便带我去取眼镜（没有时间、交通不方便）；<br>9=其他原因，请告诉我们：______________	
16. 如果收到眼镜券，你为什么没有选我们项目的眼镜？<br>（单选，答案序号写于答案列，若选 9 请将具体原因填在横线上）	0=我自己已经有眼镜，暂时不需要新眼镜；<br>1=担心戴了眼镜后视力越来越差；<br>2=我的周围基本没有人戴眼镜，担心戴了眼镜被别人笑话或者欺负；<br>3=不戴眼镜也勉强看得见，等以后看不清楚了再戴；<br>4=觉得戴着眼镜行动(或体育活动)不方便；<br>5=家长不重视我的视力情况；<br>6=爸爸、妈妈不相信可以领到免费的眼镜；<br>7=爸爸、妈妈和亲朋好友不方便带我去取眼镜（没有时间、交通不方便）；<br>8=项目组通知领眼镜的时间太短<br>9=其他原因，请告诉我们：______________	

亲爱的同学，问卷到此结束。

谢谢你支持我们的项目调查工作！祝你学习进步！

视力复查表

第一部分：视力检查

3. 是否有眼镜？ ☐ 否，结束问卷
☐ 有，未带来，结束问卷
☐ 有，带来了，查戴镜视力 ☐ 项目眼镜
☐ 非项目眼镜

裸眼视力	
**1.** 右眼	**2.** 左眼
□6/3	□6/3
□6/3.8	□6/3.8
□6/4.8	□6/4.8
□6/6	□6/6
□6/7.5	□6/7.5
□6/9.5	□6/9.5
□6/12	□6/12
□6/15	□6/15
□6/19	□6/19
□6/24	□6/24
□6/30	□6/30
□6/38	□6/38
□6/48	□6/48
□6/60	□6/60
□6/76	□6/76
□6/96	□6/96
□6/120	□6/120
□6/152	□6/152
□6/192	□6/192
□6/240	□6/240
□< 6/240	□< 6/240

戴镜视力	
**4.** 右眼	**5.** 左眼
□6/3	□6/3
□6/3.8	□6/3.8
□6/4.8	□6/4.8
□6/6	□6/6
□6/7.5	□6/7.5
□6/9.5	□6/9.5
□6/12	□6/12
□6/15	□6/15
□6/19	□6/19
□6/24	□6/24
□6/30	□6/30
□6/38	□6/38
□6/48	□6/48
□6/60	□6/60
□6/76	□6/76
□6/96	□6/96
□6/120	□6/120
□6/152	□6/152
□6/192	□6/192
□6/240	□6/240
□< 6/240	□< 6/240

二部分质检 6. 是否需要质检？ **否**，结束问卷； **是**，进入质检 ☐ ☐

验光结果：

	球镜	柱镜	轴向	最佳矫正视力
右眼	**7.**	**8.**	**9.**	**10.**
左眼	**11.**	**12.**	**13.**	**14.**

处方结果：

	球镜	柱镜	轴向	最佳矫正视力	瞳距
右眼	**15.**	**16.**	**17.**	**18.**	23.
左眼	**19.**	**20.**	**21.**	**22.**	

焦度计检测结果：

	球镜	柱镜	轴向	最佳矫正视力	瞳距
右眼	**24.**	**25.**	**26.**	**27.**	32.
左眼	**28.**	**29.**	**30.**	**31.**	

33. 焦度计检测是否和配镜处方一致？ ☐ 是，误差范围内； ☐ 否。
